# Risk Factors for Chronic Lower Back Pain among Older Workers: A Prospective Cohort Study

**DOI:** 10.1298/ptr.E10304

**Published:** 2024-11-13

**Authors:** Ryutaro MATSUGAKI, Shinya MATSUDA

**Affiliations:** 1 Department of Work Systems and Health, Institute of Industrial Ecological Sciences, University of Occupational and Environmental Health, Japan; 2 Department of Preventive Medicine and Community Health, School of Medicine, University of Occupational and Environmental Health, Japan

**Keywords:** Older worker, Chronic lower back pain, Cohort study, Risk factor

## Abstract

Objective: The purpose of this study was to identify the risk factors for the development of chronic lower back pain in older workers. Methods: This was a prospective cohort study using an Internet survey of workers aged 60–75 years, with a baseline survey conducted in September 2022 and a follow-up survey in October 2023. A total of 2257 participants who did not have chronic lower back pain in the baseline survey were included in the analysis, and the risk factors for chronic lower back pain were examined by logistic regression analysis. Results: The median age of the analyzed participants was 63.0 years, and the incidence of chronic lower back pain was 9.0%. Logistic regression analysis revealed that poor sleep habits (adjusted odds ratio [aOR]: 1.66, 95% confidence interval [CI]: 1.21–2.26), poor eating habits (aOR: 1.44, 95% CI: 1.03–2.01), no physical activity (aOR: 1.45, 95% CI: 1.00–2.09), and high stress (aOR: 1.62, 95% CI: 1.13–2.32) were significantly associated with the occurrence of chronic lower back pain. Conclusion: A comprehensive multidisciplinary collaboration program incorporating the assessment and management of lifestyle habits and mental health issues should be developed and implemented to prevent chronic lower back pain in older workers.

## Introduction

Chronic lower back pain is widely recognized as a typical health disorder in the working population, with a reported prevalence of approximately 20%^1)^. In addition, the incidence of chronic lower back pain tends to increase with age^2)^. This suggests that the problem will become even more significant in the future as the aging population increases. The prevalence of musculoskeletal pain in older workers not only affects an individual’s quality of life but also causes socioeconomic problems, such as disability pension benefits and unemployment^3^^,^^4)^. Therefore, the development and implementation of measures to prevent chronic lower back pain in older workers is of critical importance from a public health perspective.

Several factors have been identified as risk factors for chronic lower back pain in community-dwelling older adults. Previous cohort studies have shown that factors such as body mass index (BMI) and occupational biomechanical exposure are associated with the incidence of chronic lower back pain in older adults^5^^,^^6)^. Additionally, other cohort studies have also reported that factors such as sex and educational background are related to the persistence of lower back pain^7)^. Furthermore, prospective cohort studies and recent meta-analyses have indicated that physical activity in leisure time contributes to a reduced risk of chronic lower back pain in middle-aged and older individuals. These findings suggest that these factors should be considered as preventive measures for chronic lower back pain in older workers.

Although extensive knowledge of risk factors for chronic lower back pain in older adults including non-workers has been accumulated, whether these findings can be applied to older worker populations remains unclear. The socioeconomic conditions and lifestyle habits of community-dwelling older adults and older workers may differ, potentially leading to different risk factors. Moreover, work-related factors, such as the nature of the job^8–10)^, have been suggested to be associated with chronic lower back pain in workers. However, these work-related factors have rarely been considered in studies on older adults.

The objective of this study was to explore the risk factors for chronic lower back pain among older workers and obtain foundational data to inform preventive strategies for this population.

## Methods

### Study design and participants

This was a prospective cohort study using data from an Internet survey of workers aged 60–75 years in the tertiary industry in Japan. This study was designed to examine occupational accidents and health issues among older workers.

We conducted our baseline study in September 2022^11)^. For the baseline survey, we sent questionnaires to those who responded that they were working at the time of the survey panel registration among the survey panels held by the Cross Marketing Corporation (Tokyo, Japan). The exclusion criteria were (1) patients who were not working at the time of the survey, (2) who were aged <60 years or >76 years, (3) who were self-employed or a family member of a self-employed person engaging without pay in a business operated by that person, (4) who were not engaged in the tertiary industry, and (5) who gave incorrect answers to fraud detection questions (questions requiring respondents to select the third highest number among the 5 numbers). The survey was terminated when responses were collected from 5000 respondents who met the inclusion criteria.

A follow-up survey was conducted in October 2023 for the 5000 participants for whom data were collected in the baseline survey. A total of 3113 individuals responded to the follow-up survey, and 2873 individuals were included in the study. We excluded those who provided fraudulent responses (n = 240). Therefore, a total of 2257 participants from the baseline survey were included in the analysis, excluding those with chronic lower back pain (n = 616).

### Survey items

The study included age at baseline (60–64, 65–69, and 70–75 years), sex, BMI (<18.5, 18.5–24.9, and ≥25.0 kg/m^2^), socioeconomic factors, lifestyle, mental health, and the presence of chronic lower back pain at follow-up.

Socioeconomic factors, including educational background (middle school/high school, vocational/junior college/technical school, and college/graduate school), subjective economic status, employment status (regular or non-regular employment), job type (mainly desk work, mainly work talking to people, and mainly physical work), work frequency (<3 days/week, 3–4 days/week, and ≥5 days/week), and industry (wholesale/retail, medical/welfare, and other), were surveyed. The subjective economic situation was defined as “difficult” if the respondent answered “very difficult” or “somewhat difficult” to the question “How do you feel about your current living situation from an economic viewpoint?” The respondents who answered “normal,” “somewhat comfortable,” or “very comfortable” were defined as “not difficult.”

Lifestyle habits were investigated in terms of sleep, physical activity, dietary habits, alcohol consumption, and smoking history, based on a standard questionnaire used for health examinations in Japan^12^^,^^13)^. Poor sleep habits were defined if the respondent answered no to the “Have enough sleep” item. Physical activity was defined as “Engaging in a walk or an equivalent physical activity for at least 1 hour a day in your daily life” or “Doing exercise to sweat lightly for over 30 minutes a time, 2 times weekly, for over a year.” No physical activity was defined if the respondent answered no to either of these questions. Smoking habit was defined as “yes” to the item “Currently smoking cigarettes on a regular basis.” An alcohol consumption habit was defined when a participant answered yes to the item “Drink alcohol (sake, shochu, beer, Western liquor, etc.) occasionally or daily.” Poor eating habits were defined if the participant answered yes to any of the following questions: “Eat faster than usual,” “Have dinner within 2 hours before going to bed at least 3 times a week”, “Have a snack a night snack other than the 3 meals,” and “Skip breakfast at least 3 times a week.”

Psychological distress was assessed using the Kessler Psychological Distress Scale-six items (K6), developed by Kessler et al., to screen for psychiatric disorders, such as depression and anxiety disorders^14^^,^^15)^. In this study, the presence of psychological distress was defined as a score of 5 or higher on the K6^16)^.

Chronic lower back pain was defined as “yes” to the question “Do you have lower back pain that has persisted for more than 3 months?”

### Statistical analysis

A binomial logistic regression analysis was conducted with chronic lower back pain as the objective variable to examine factors associated with chronic lower back pain. First, crude odds ratios (ORs) and 95% confidence intervals (CIs) were calculated using a univariate model in which age, sex, BMI, socioeconomic factors, lifestyle, and psychological stress responses were each used as explanatory variables. Then, adjusted ORs (aORs) and 95% CIs were calculated using multivariate models in which age, sex, BMI, socioeconomic factors, lifestyle, and psychological stress responses were all simultaneously cast as explanatory variables. In this study, we selected explanatory variables that were considered potential risk factors for chronic lower back pain based on insights from previous research and discussions among the research team. These variables were included in the model using the forced entry method.

Stata MP/18.0 (StataCorp LLC, College Station, TX, USA) was used for all statistical analyses, with a significance level of 5%.

### Supportive analysis

Factors contributing to chronic lower back pain may differ between workers engaged primarily in physical labor and other types of work. Therefore, a multivariable binomial logistic regression analysis stratified by mainly physical and mainly non-physical work (mainly desk work and work involving communication) was conducted as a supportive analysis. The explanatory variables for the multivariable binomial logistic regression were the same as those for the main analysis except for the job type.

### Ethical considerations

This study was conducted with the approval of the Ethics Committee of the University of Occupational and Environmental Health Sciences (approval number: R4-031). In addition, consent to participate in the study was obtained from all respondents using a check box provided on the questionnaire response screen.

## Results

Table 1 shows the attributes of the 2257 analyzed participants. The median age of the participants was 63.0 years (interquartile range: 61.0–66.0 years), and the proportion of females was 45.3% (1023/2257). The incidence of chronic lower back pain was 9.0% (203/2257). The group with chronic lower back pain had poorer sleep habits (with chronic lower back pain vs. without chronic lower pain; 61.1% vs 46.4%), a higher proportion of lacking physical activity (79.8% vs 72.5%), poorer eating habits (73.9% vs 65.1%), and higher stress levels (25.6% vs 15.8%) in contrast to the group without chronic lower back pain.

**Table 1.** Characteristics of participants

	Chronic lower back pain	
	Without (n = 2054)	With (n = 203)
Age (years)		
60–64	1386 (67.5%)	144 (70.9%)
65–69	483 (23.5%)	37 (18.2%)
70–75	185 (9.0%)	22 (10.8%)
Female sex	932 (45.4%)	91 (44.8%)
Body mass index (kg/m^2^)		
<18.5	243 (11.8%)	19 (9.4%)
18.5–24.9	1405 (68.4%)	145 (71.4%)
≥25.0	406 (19.8%)	39 (19.2%)
Educational background		
Junior high school/high school	531 (25.9%)	57 (28.1%)
Vocational school/junior college/technical college	423 (20.6%)	43 (21.2%)
University/graduate school	1100 (53.6%)	103 (50.7%)
Subjective economic situation (difficult)	596 (29.0%)	67 (33.0%)
Employment status (non-regular employment)	1202 (58.5%)	119 (58.6%)
Job description		
Mainly desk work	924 (45.0%)	93 (45.8%)
Mainly work talking to people	552 (26.9%)	53 (26.1%)
Mainly physical work	578 (28.1%)	57 (28.1%)
Work frequency (days per week)		
<3	158 (7.7%)	14 (6.9%)
3–4	488 (23.8%)	62 (30.5%)
≥5	1408 (68.5%)	127 (62.6%)
Industry classification		
Wholesale, retail	503 (24.5%)	42 (20.7%)
Medical, healthcare, and welfare	378 (18.4%)	44 (21.7%)
Others	1173 (57.1%)	117 (57.6%)
Sleep habit (poor)	954 (46.4%)	124 (61.1%)
Physical activity (no)	1489 (72.5%)	162 (79.8%)
Eating habit (poor)	1337 (65.1%)	150 (73.9%)
Drinking habit (yes)	1034 (50.3%)	107 (52.7%)
Smoking habit (yes)	468 (22.8%)	43 (21.2%)
Psychological distress (K6 ≥5)	325 (15.8%)	52 (25.6%)

K6, Kessler Psychological Distress Scale-six items

Table 2 shows the results of the logistic regression analysis with chronic lower back pain as the objective variable. In the multivariate model, the results showed that poor sleep habits (aOR: 1.66, 95% CI: 1.21–2.26, p = 0.002), poor eating habits (aOR: 1.44, 95% CI: 1.03–2.01, p = 0.032), no physical activity (aOR: 1.45, 95% CI: 1.00–2.09, p = 0.048), and high stress (aOR : 1.62, 95% CI: 1.13–2.32, p = 0.008) were significantly associated with the occurrence of chronic lower back pain. On the other hand, age, sex, BMI, and socioeconomic factors showed no association with chronic lower back pain.

**Table 2.** Logistic regression analysis results with chronic lower back pain as the dependent variable

	Univariate model				Multivariate model			
	Crude OR	95% CI		p-value	Adjusted OR	95% CI		p-value
Age (years)								
60–64	Reference				Reference			
65–69	0.74	0.51	1.07	0.112	0.77	0.52	1.14	0.188
70–75	1.14	0.71	1.84	0.577	1.25	0.75	2.06	0.391
Female sex	0.98	0.73	1.31	0.881	0.88	0.61	1.28	0.520
Body mass index (kg/m^2^)								
<18.5	0.76	0.46	1.25	0.274	0.72	0.43	1.21	0.220
18.5–24.9	Reference				Reference			
≥25.0	0.93	0.64	1.35	0.704	0.86	0.59	1.27	0.454
Educational background								
Junior high school/high school	Reference				Reference			
Vocational school/junior college/technical college	0.95	0.62	1.44	0.797	0.94	0.61	1.44	0.766
University/graduate school	0.87	0.62	1.23	0.431	0.85	0.59	1.24	0.396
Subjective economic situation (difficult)	1.21	0.89	1.64	0.235	0.97	0.70	1.35	0.858
Employment status (non-regular employment)	1.00	0.75	1.35	0.978	0.90	0.63	1.27	0.540
Job description								
Mainly desk work	Reference				Reference			
Mainly work talking to people	0.95	0.67	1.36	0.794	0.90	0.62	1.30	0.584
Mainly physical work	0.98	0.69	1.38	0.908	0.85	0.58	1.24	0.400
Work frequency (days per week)								
<3	Reference				Reference			
3–4	1.43	0.78	2.63	0.245	1.36	0.73	2.52	0.333
≥5	1.02	0.57	1.81	0.952	0.83	0.44	1.55	0.557
Industry classification								
Wholesale, retail	0.84	0.58	1.21	0.343	0.86	0.59	1.25	0.423
Medical, healthcare, and welfare	1.17	0.81	1.68	0.408	1.16	0.79	1.71	0.439
Others	Reference				Reference			
Sleep habit (poor)	1.81	1.35	2.43	0.000	1.66	1.21	2.26	0.002
Physical activity (no)	1.50	1.05	2.14	0.026	1.45	1.00	2.09	0.048
Eating habit (poor)	1.52	1.10	2.10	0.012	1.44	1.03	2.01	0.032
Drinking habit (yes)	1.10	0.82	1.47	0.520	1.11	0.82	1.51	0.488
Smoking habit (yes)	0.91	0.64	1.30	0.603	0.85	0.59	1.23	0.396
Psychological distress (K6 ≥5)	1.83	1.31	2.57	0.000	1.62	1.13	2.32	0.008

OR, odds ratio; CI, confidence interval; K6, Kessler Psychological Distress Scale-six items

In the multivariable binomial logistic regression analysis stratified by job type, sleep habits (aOR: 3.20, 95% CI: 1.66–6.17, p = 0.001) and poor eating habits (aOR: 2.13, 95% CI: 1.04–4.38, p = 0.039) were shown to be associated with the occurrence of chronic lower back pain in mainly manual workers. On the other hand, in mainly non-manual workers, lacking physical activity (aOR: 1.73, 95% CI: 1.10–2.73, p = 0.019) and a high stress level (aOR: 1.69, 95% CI: 1.09–2.61, p = 0.018) were associated with the occurrence of chronic lower back pain (Table S1).

## Discussion

We conducted a prospective cohort study using Internet survey techniques. We found that undesirable lifestyle habits (poor sleep and eating habits and no physical activity) and high psychological stress were associated with the occurrence of chronic lower back pain among older workers. Our findings suggest the need for lifestyle-based health management and mental health measures to prevent chronic lower back pain among older workers. This finding provides important insights for older workers, the proportion of which has been increasing worldwide in recent years.

Undesirable sleep habits were associated with the occurrence of chronic lower back pain in older workers. The results of this study suggest that the findings of cohort studies showing that sleep problems are associated with musculoskeletal pain in workers may be applicable to older workers^17–19)^. Although the mechanisms by which sleep problems influence the development of pain are inconclusive, based on the results of a recent systematic review and meta-analysis^20)^, sleep problems may influence the development of chronic lower back pain by lowering the pain threshold and pain tolerance. Our findings suggest that sleep problems are not only a common health problem in the elderly^21^^,^^22)^ but also one of the issues that need to be addressed when considering measures to prevent chronic lower back pain in older workers.

Physical inactivity was also associated with the occurrence of chronic lower back pain in older workers. Similar results to those of the present study were confirmed in a systematic review and meta-analysis based on 7 cohort study articles, indicating that moderate-intensity physical activity has the potential to reduce the risk of lower back pain^23)^. However, the health effects of physical activity have been suggested to differ between leisure time physical activity and occupational physical activity^24^^,^^25)^, and a prospective cohort study in Norway found that high physical activity in the workplace was associated with an increased risk of chronic lower back pain^26)^. This suggests that higher physical activity at work increases the risk of chronic lower back pain, but whether this is applicable to older workers has not been established. However, the results of this study suggest that interventions focusing on the amount of physical activity during leisure time are needed to prevent chronic lower back pain in older workers.

Poor dietary habits were found to be associated with the occurrence of chronic lower back pain in older workers, but a high BMI (>25 kg/m^2^) was not associated with the occurrence of chronic lower back pain. This finding differs from previous studies suggesting that a high BMI is associated with lower back pain in workers^27–31)^. These previous studies included younger workers, which may have contributed to the inconsistency of the results with the present study. In older workers, a higher BMI means that muscle mass is maintained, which may mitigate the negative effect of increased body weight on lower back pain. Although the relationship between BMI and chronic lower back pain in older workers requires further investigation, our findings suggest that the prevention of chronic lower back pain in older workers should focus on the quality of dietary habits rather than simple weight control.

In addition to lifestyle factors, this study found that mental health problems were associated with the occurrence of chronic lower back pain in a population of older workers. Previous cohort studies have shown that mental health problems are associated with the transition from acute to chronic lower back pain^32^^,^^33)^, and our findings emphasize the importance of mental health assessment and management in chronic lower back pain prevention programs for older workers. In addition, recent meta-analyses have shown that psychosocial factors, such as job control and decision-making authority, are also associated with chronic lower back pain^34)^. In light of these findings, it is important to focus on psychosocial factors underlying mental health problems, as well as mental health problems themselves, in the prevention of chronic lower back pain.

However, analyses stratified by job content suggested that risk factors may differ between manual and non-manual workers. Specifically, sleep and diet were associated with chronic lower back pain in the predominantly physical work group, whereas physical activity and mental health problems were associated with chronic lower back pain in the primarily non-physical work group. These findings suggest that risk factors for chronic lower back pain may differ between job types, and therefore effective strategies for preventing lower back pain may also differ between job types. However, these findings are based on ancillary analyses and need to be validated in future studies to clarify causal relationships.

This study had several limitations. First, the physical work environment, workload, and posture, which are suggested to be related to lower back pain^10^^,^^35^^,^^36)^, were not considered in this study. The participants were older workers in the tertiary industry, where even mainly physical work might involve lighter workloads and well-maintained work environments suitable for older workers. As a result, the relationship between job description and chronic lower back pain may be underestimated. Additionally, whether the findings of this study can be applied to workers in the primary and secondary industries is unclear. Second, the physical capacity of the participants, such as muscle strength, was not taken into account in this study. Although the relationship between physical capacity and chronic lower back pain in older adults has not been clearly established, physical capacity is known to be closely related to age^37)^. Therefore, the omission of this variable may have influenced the results of the study. Future research should include assessments of physical capacity to gain a more complete understanding of the risk factors for chronic lower back pain in older workers. Third, this study relied on self-reported data, which may be subject to recall bias. Specifically, chronic lower back pain was defined as “lower back pain lasting more than 3 months.” Participants with longer duration of pain, such as 6 months, were expected to report accurately. However, for those with pain lasting only 3 months, there is a risk of recall misclassification, potentially leading to incorrect reporting of nothing as chronic lower back pain. Such incidental misclassification could lead to an underestimation of the associations between various factors and chronic lower back pain. Finally, although sleep habits, eating habits, and physical activity were found to be associated with the occurrence of chronic lower back pain, it is not possible to conclude from the results of this study whether interventions in these areas contribute to preventing the occurrence of chronic lower back pain. Future intervention studies should be conducted to verify whether lifestyle guidance can prevent the occurrence of chronic lower back pain.

## Conclusion

This study identified poor sleep habits, lack of physical activity, inappropriate dietary habits, and high stress as risk factors for chronic lower back pain in older workers. The development and implementation of a comprehensive intervention strategy involving multidisciplinary collaboration (such as occupational physicians, occupational health nurses, dietitians, psychologists, and physical therapists) focusing on the assessment and management of lifestyle habits and mental health is essential to prevent chronic lower back pain in older workers.

## Funding

This study was supported by the Japan Small and Medium-Sized Enterprise Welfare Foundation, the Japanese Society of Physical Therapy (SP2022-031), and JSPS KAKENHI (24K20394).

## Conflicts of Interest

The authors declare no conflicts of interest associated with this manuscript.

## Supplementary Material

Table S1. Results of multivariable logistic regression analysis stratified by job types with chronic lower back pain as the dependent variable.
