# Supplementary material for: Risk Factors for Chronic Lower Back Pain among Older Workers: A Prospective Cohort Study
Source: Phys Ther Res. 2024 Nov 13;27(3):151–7. doi: 10.1298/ptr.E10304 (PMC11756560; doi:10.1298/ptr.E10304)
Supplement: Table S1. — Results of multivariable logistic regression analysis stratified by job types with chronic lower back pain as the dependent variable. [file ptr-27-151-s01.pdf]

Table S1. Results of multivariable logistic regression analysis stratified by job types with chronic lower back pain as the dependent variable

	Mainly manual work				Mainly not manual work*			
	adjusted OR	95% CI		p-value	adjusted OR	95% CI		p-value
Age (years)								
60-64	Reference				Reference			
65-69	0.60	0.27	1.33	0.211	0.82	0.52	1.29	0.396
70-75	1.12	0.44	2.87	0.815	1.30	0.71	2.40	0.395
Female sex	0.99	0.49	2.00	0.974	0.83	0.53	1.30	0.419
Body mass index (kg/m^2)								
<18.5	0.45	0.17	1.24	0.123	0.90	0.49	1.65	0.743
18.5-24.9	Reference				Reference			
≥25.0	0.71	0.32	1.58	0.400	0.93	0.60	1.44	0.733
Educational background								
Junior high school/high school	Reference				Reference			
Vocational school/junior college/technical college	0.87	0.42	1.84	0.723	0.96	0.56	1.64	0.868
University/graduate school	0.78	0.39	1.57	0.489	0.84	0.54	1.33	0.464
Subjective economic situation (difficult)	1.47	0.81	2.69	0.206	0.81	0.54	1.23	0.330
Employment status (non-regular employment)	0.85	0.40	1.80	0.677	0.92	0.62	1.36	0.661
Work frequency (days per week)								
<3	Reference				Reference			
3-4	0.68	0.22	2.04	0.487	1.82	0.85	3.92	0.125
≥5	0.52	0.17	1.58	0.251	0.99	0.46	2.14	0.989
Industry classification								
Wholesale, retail	0.66	0.24	1.81	0.416	0.87	0.58	1.31	0.521
Medical, healthcare, and welfare	1.32	0.69	2.52	0.400	1.08	0.65	1.78	0.773

Others	Reference				Reference			
Sleeping habit (poor)	3.20	1.66	6.17	0.001	1.33	0.92	1.92	0.124
Physical activity (no)	1.01	0.53	1.92	0.983	1.73	1.10	2.73	0.019
Eating habit (poor)	2.13	1.04	4.38	0.039	1.30	0.88	1.90	0.183
Drinking habit (yes)	1.18	0.65	2.12	0.587	1.11	0.78	1.60	0.560
Smoking habit (yes)	0.58	0.26	1.26	0.169	0.96	0.63	1.46	0.848
Psychological distress (K6≧5)	1.52	0.79	2.93	0.207	1.69	1.09	2.61	0.018

CI, confidence interval; K6, Kessler Psychological Distress Scale-six items; OR, odds ratio.

* Mainly desk work and work involving communication
